# Substrate specificity and ecological significance of PstS homologs in phosphorus uptake in marine *Synechococcus* sp. WH8102

**DOI:** 10.1128/spectrum.02786-23

**Published:** 2024-01-05

**Authors:** Pramita Ranjit, Deepa Varkey, Bhumika S. Shah, Ian T. Paulsen

**Affiliations:** 1 School of Natural Sciences, Macquarie University, Sydney, Australia; 2 ARC Centre of Excellence in Synthetic Biology, Macquarie University, Sydney, Australia; University of Minnesota Twin Cities, St. Paul, Minnesota, USA

**Keywords:** phosphate binding protein (PstS), *Synechococcus*, phosphate stress, high-affinity, differential scanning fluorimetry (DSF)

## Abstract

Phosphorus, a vital macronutrient, often limits primary productivity in marine environments. Marine *Synechococcus* strains, including WH8102, rely on high-affinity phosphate-binding proteins (PstS) to scavenge inorganic phosphate in oligotrophic oceans. However, WH8102 possesses three distinct PstS homologs whose substrate specificity and ecological roles are unclear. The three PstS homologs were heterologously expressed and purified to investigate their substrate specificity and binding kinetics. Our study revealed that all three PstS homologs exhibited a high degree of specificity for phosphate but differed in phosphate binding affinities. Notably, PstS1b displayed nearly 10-fold higher binding affinity (*K*_D_ = 0.44 µM) compared to PstS1a (*K*_D =_ 3.3 μM) and PstS2 (*K*_D =_ 4.3 μM). Structural modeling suggested a single amino acid variation in the binding pocket of PstS1b (threonine instead of serine in PstS1a and PstS2) likely contributed to its higher Pi affinity. Genome context data, together with the protein biophysical data, suggest distinct ecological roles for the three PstS homologs. We propose that PstS1b may be involved in scavenging inorganic phosphorus in oligotrophic conditions and that PstS1a may be involved in transporting recycled phosphate derived from organic phosphate cleavage. The role of PstS2 is less clear, but it may be involved in phosphate uptake when environmental phosphate concentrations are transiently higher. The conservation of three distinct PstS homologs in *Synechococcus* clade III strains likely reflects distinct adaptations for P acquisition under varying oligotrophic conditions.

**IMPORTANCE:**

Phosphorus is an essential macronutrient that plays a key role in marine primary productivity and biogeochemistry. However, intense competition for bioavailable phosphorus in the marine environment limits growth and productivity of ecologically important cyanobacteria. In oligotrophic oceans, marine *Synechococcus* strains, like WH8102, utilize high-affinity phosphate-binding proteins (PstS) to scavenge inorganic phosphate. However, WH8102 possesses three distinct PstS homologs, with unclear substrate specificity and ecological roles, creating a knowledge gap in understanding phosphorus acquisition mechanisms in picocyanobacteria. Through genomic, functional, biophysical, and structural analysis, our study unravels the ecological functions of these homologs. Our findings enhance our understanding of cyanobacterial nutritional uptake strategies and shed light on the crucial role of these conserved nutrient uptake systems in adaptation to specific niches, which ultimately underpins the success of marine *Synechococcus* across a diverse array of marine ecosystems.

## INTRODUCTION

Phosphorus (P) is an essential macronutrient vital in synthesizing numerous biomolecules including DNA, RNA, ATP, and nucleotides (1). Many oceanic regions have been recorded to be P limited (2–4), which in turn limits the growth of the most abundant and ecologically significant marine picocyanobacteria, *Synechococcus* and *Prochlorococcus* (5, 6)*,* in these areas. Dissolved inorganic phosphate concentrations, considered a preferable P source for marine picocyanobacteria compared to dissolved organic phosphorus, are usually found in lower nanomolar ranges in the oligotrophic oceans (3). Hence, there is likely fierce competition for bioavailable P *in situ*. Given the importance of P and its limitation in the oligotrophic oceans, marine picocyanobacteria utilize several strategies to survive the low P conditions, including scavenging inorganic P using high-affinity periplasmic phosphate binding proteins (PstS) (5, 7), using organic P following hydrolysis via alkaline phosphatase activity (7, 8), high phosphate uptake rates (9), and replacing membrane phospholipids for sulfolipids (10).

The importance of high-affinity capture of inorganic P (Pi) via PstS in marine picocyanobacteria has been highlighted in several comparative genomics (11, 12) and metagenomic studies (13) which have found genomes encoding multiple copies of the *pstS* gene, especially in picocyanobacterial ecotypes/clades inhabiting the low-P regions. As aforementioned, high-affinity phosphate transport in marine picocyanobacteria is facilitated by a periplasm located substrate-binding protein (PstS) and associated membrane-bound ABC transporters (PstCAB) (14, 15) which is regulated by the PhoBR two-component system, which in response to P limitation increases transcription of phosphate acquisition genes (1, 16, 17). Genome predictions in many freshwater cyanobacterial strains depicted that more than half contain duplicate Pst transporters. For example, in *Synechocystis* sp. 6803, two gene clusters encoding Pi ABC transporters (*pst*1 and *pst*2) with three associated phosphate-binding proteins (PBP) are present (18). In contrast, most marine picocyanobacterial strains encode a single Pst transporter, but the number of PstS homologs is variable. For example, *Synechococcus* sp. WH8102 (hereafter known as WH8102), belonging to clade III that typically inhabits low-P marine environments, encodes a single Pst transporter and three PstS homologs in addition to a closely related predicted phosphate-binding protein SphX (19, 20). Transcriptomic studies in WH8102 have shown that all PBP homologs were upregulated under P stress conditions (1).

In general, ABC transporters play a critical role in translocating nutrients from the surrounding environment into cells. The substrate binding proteins (SBPs) serve as a key component of ABC uptake transporters as the primary determinants of substrate specificity (21, 22). It has been observed that some ABC transporters can work in conjunction with multiple SBPs, each with a distinct substrate specificity, likely functioning to increase substrate range at a minimal cost (23). For example, the CbcWV transporter in *Pseudomonas* sp. interacts with multiple SBPs, namely, CbcX, CaiX, and BetX, involved in the uptake of choline, carnitine, and betaine, respectively (24). Similarly, the HisQMP transporter in *S. typhimurium* employs two distinct SBPs, ArgT, and HisJ, which have overlapping specificities for histidine, arginine, lysine, and ornithine (25). It is unknown if the three PstS homologs [hereafter known as PstS1a (SYNW01018), PstS1b (SYNW01815), and PstS2 (SYNW02507)] in WH8102 have different ligand specificities, i.e., if they bind to other P-containing ligands which would enable them to scavenge various P sources or if they have different binding affinities for phosphate, thereby optimizing transport over an extended range of P concentration.

Therefore, in this study, we examined all three PstS homologs in WH8102 isolated from the Sargasso Sea, an oligotrophic region of the Atlantic Ocean (26), to understand their substrate specificity and binding kinetics as well as explore their ecological and physiological roles. We demonstrate that all three copies of PstS are highly specific for phosphate but suggest distinct ecological roles for each of the homologs.

## RESULTS AND DISCUSSION

### PstS homologs are potentially eco-paralogs with distinct ecological roles to P stress response

**Fig 1:**
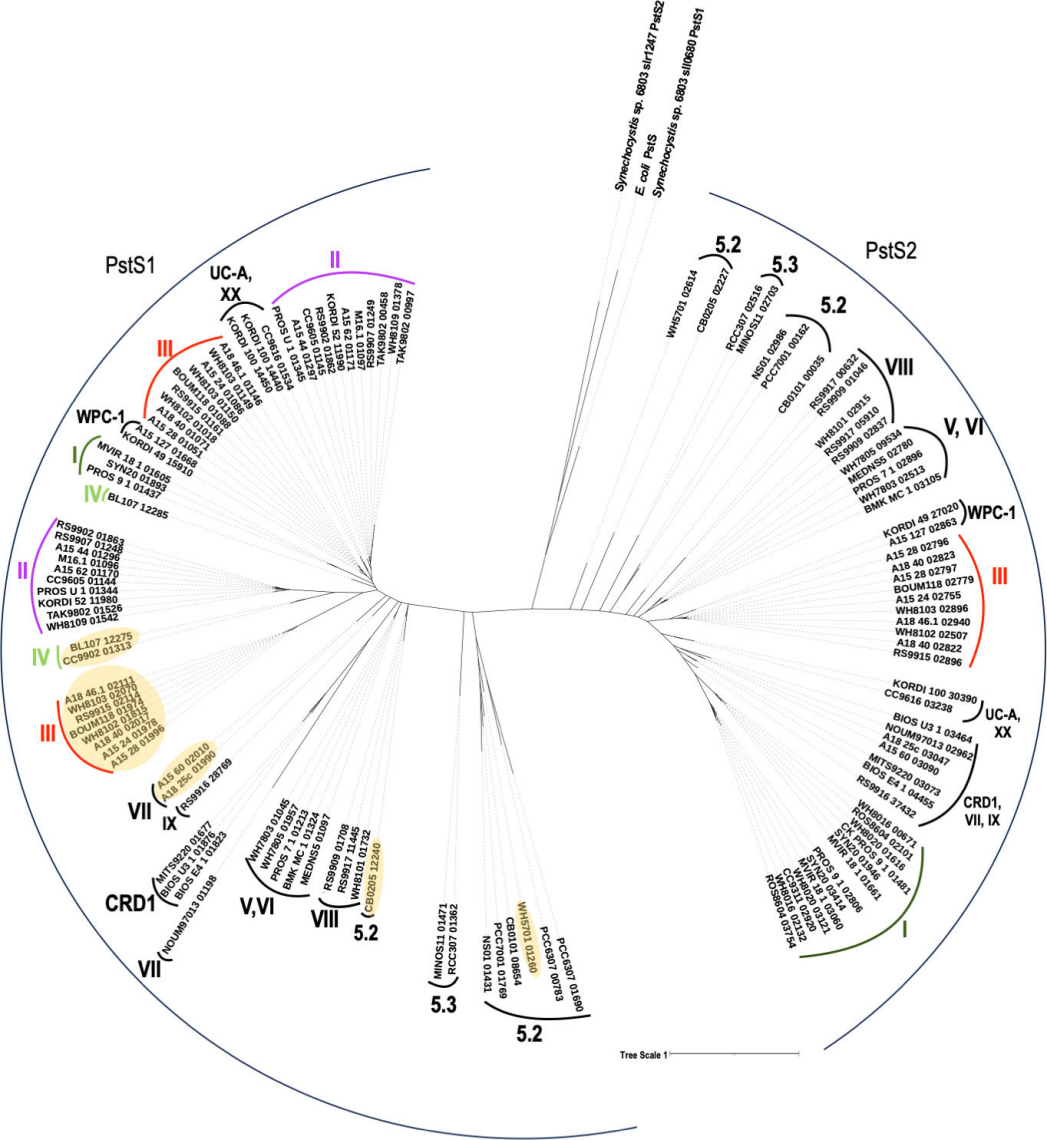
Phylogenetic analyses of *Synechococcus* PstS sequences. Amino acid sequences of *Synechococcus* PstS1 and PstS2, belonging to cluster CK_00043821 and CK_00000023, were aligned using MAFFT (28). The phylogenetic tree was inferred using IQ-Tree (29) and visualized using iTOL (30). Cyanorak cluster designations (27) are used to denote the gene numbers. Protein clusters are labeled with *Synechococcus* clade designation along with PstS1 and PstS2 lineages. *Synechococcus* PstS1 with threonine residue in the binding pocket are highlighted in yellow (see Fig. 4 for further details).

A phylogenetic tree comprising all *Synechococcus* PstS (PstS1 and PstS2) protein sequences present in the Cyanorak database (27) along with PstS protein sequences from model freshwater cyanobacteria *Synechocystis* sp. 6803 and *E. coli* as an outgroup is depicted in Fig. 1. The phylogenetic tree reveals that PstS1 and PstS2 proteins delineate into two distinct clusters, corresponding to Cyanorak clusters CK_00043821 and CK_00000023, respectively. Within each cluster, the proteins fall into *Synechococcus* clade-specific subfamilies.

The *pstS1* gene is conserved in all *Synechococcus* strains except for a few strains belonging to clade I (CC9311, WH8020, ROS8604, and WH8106), indicating strong evolutionary pressure for its maintenance. In WH8102, two genes *pstS1a* (SYNW1018) and *pstS1b* (SYNW1815), both belonging to the same cluster, have been annotated as *pstS1* in the Cyanorak database. Given the highly streamlined genomes of marine picocyanobacteria (5), it is likely that one of these genes has been vertically inherited while the other one is a result of a recent gene duplication event and, hence, are gene paralogs that have been retained likely because they have distinct physiological roles. Duplicated genes are usually reduced to pseudogenes and eventually deleted from the genome if they do not confer any selective advantage (31). Similarly, *pstS2* (SYNW2507) is also present in almost all *Synechococcus* strains except for clade II and IV strains. It is possible that the multiple *pstS* genes are ecoparalogs, i.e., they perform the same general function but most likely function under different conditions. Ecoparalogs have been observed in bacteria such as *Salinibacter ruber*, which has halophilic proteins that allow them to function over a wide range of salinity (32). Likewise, multiple copies of *pstS* genes may enable marine picocyanobacteria to uptake P and survive under a wide range of phosphorus regimes, especially in oligotrophic marine environments.

**Fig 2:**
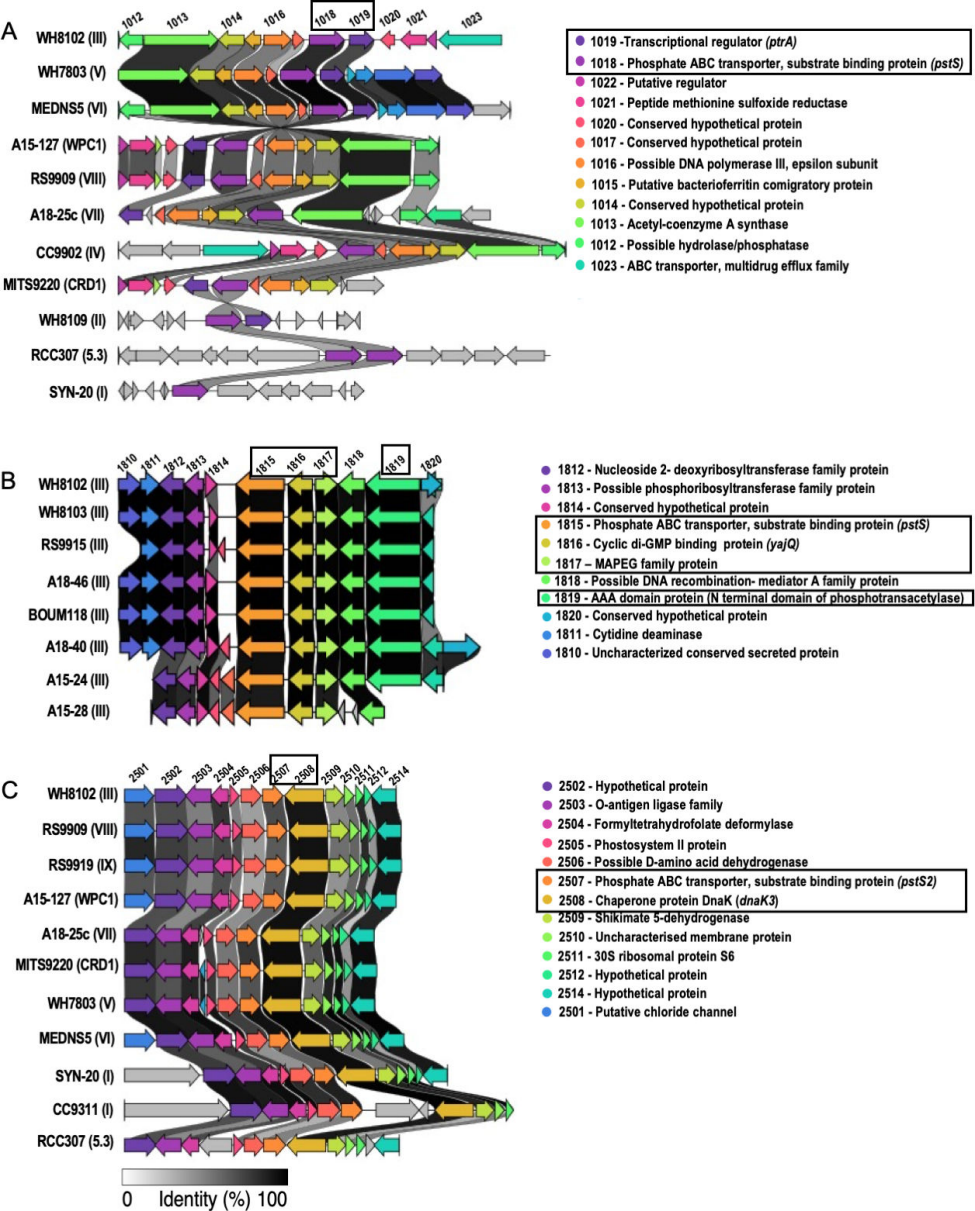
Genomic context analysis of PstS homologs in WH8102 and other selected *Synechococcus* clade representatives. Visualization of conservation of (**A**) *pstS1a,* (**B**) *pstS1b,* and (**C**) *pstS2* in representative *Synechococcus* strains. *Synechococcus* clade information for each representative strain is shown in a bracket. Genes are highlighted by color. The numbers above represent the gene locus tag in WH8102, for which respective gene product details are shown next to color legends. Each *pstS* homolog in WH8102 and the features noted in the text are shown in a box. The gene-neighborhood diagram was made using Clinker (34).

Genome context analysis of the *pstS* genes in marine *Synechococcus* WH8102 provides clues regarding their possible ecological roles (Fig. 2). In WH8102, the *pstS1a* gene is located upstream of *ptrA*, encoding a transcriptional regulator involved in regulating the expression of phosphatases for scavenging organic P under P stress conditions and is partially co-transcribed with *pstS1a* (33). Comparison with other *Synechococcus* strains in the Cyanorak database that contain *pstS1a* shows that this arrangement is conserved in 77% of these strains (Fig. 2A). The genome context analysis for *pstS1a* suggests a possible role in the transport of Pi liberated from organic P via the action of these phosphatases. Previous studies have suggested a two-tiered transcriptional response to P stress, with the first level involving Pi scavenging during early P stress, which is induced by PhoB and involves the expression of *pstS* genes along with an increase in *ptrA* levels. Increased *ptrA* levels then lead to a second-tiered response characterized by scavenging organic P (33).

In WH8102, *pstS1b* is located adjacent to genes encoding putative signaling molecules and/or stress response genes, such as cyclic di-GMP binding protein (SYNW1816), membrane-associated glutathione metabolism (MAPEG) protein (SYNW1817), and a AAA phosphoacetyltransferase domain protein (SYNW1819) (Fig. 2B). This arrangement is conserved in all *Synechococcus* clade III strains in the Cyanorak database. Generally, in bacteria, cyclic di-GMP is a universal signaling molecule governing survival strategy (35), while the MAPEG protein protects against oxidative and metabolic stress, especially heat and cold temperature stress (36). Similarly, phosphoacetyltransferase catalyzes the production of acetyl phosphate, which is known to act as a phosphodonor for response regulators (37). Hence, the occurrence of *pstS1b* near these genes suggests a role in survival under stress conditions such as P stress.

The WH8102 *pstS2* gene is located close to *DNAK3*, encoding a chaperone heat shock protein whose expression is induced by DNA-damaging agents (38). This arrangement is conserved in all *Synechococcus* strains containing *pstS2* except for selected clade I strains (CC9311, WH8020, ROS8604, and WH8016) where *DNAK3* is located one to three genes downstream of the *pstS2* gene (Fig. 2C).

### Ligand binding assays reveal PstS1b has the strongest affinity for phosphate

Genes encoding *pstS1a*, *pstS1b*, and *pstS2,* from WH8102, were cloned into pOPIN-F (*pstS1a*, *pstS1b*) and pOPIN-S (*pstS2*) vector via ligation-independent cloning to incorporate a His-tag and heterologously expressed in *E. coli* Lemo21(DE3) cells. All three proteins were successfully purified via immobilized metal affinity chromatography (IMAC) as a monodisperse product in >95% purity, verified using SDS-PAGE, and displayed a monomeric state in solution determined using analytical size exclusion chromatography (SEC) (Fig. S1). To assess the substrate specificity of all PstS proteins, a thermal shift resulting from protein-ligand interaction was determined using differential scanning fluorimetry (DSF). The change in melting temperature (Δ T_M_) of each of the proteins in the presence of different organic and inorganic P sources, including phosphate, phosphite, β-glycerophosphate, and sodium tripolyphosphate, is shown in Table 1. All PstS proteins showed the highest thermal stability and, hence, the highest shift in melting temperature in the presence of phosphate (Δ T_M_ 7.5°C–16°C) as compared to other P sources tested (Table 1; Fig. S2). A minor thermal shift was observed in the presence of tripolyphosphate (Δ T_M_ 3°C–8°C), with the highest shift observed for PstS1b.

**TABLE 1:** Change in melting temperature (Δ Tm) of PstS proteins in the presence of different phosphorus sources

	Phosphate (Δ T_M_ °C)	Phosphite (Δ T_M_ °C)	β-Glycerophosphate (Δ T_M_ °C)	Sodium tripolyphosphate (Δ T_M_ °C)
PstS1a	7.5 ± 0.20	1.7 ± 0.0	0.2 ± 0.07	2.5 ± 0.17
PstS1b	16 ± 0.15	4 ± 0.05	1.4 ± 0.20	8 ± 0.15
PstS2	8 ± 0.0	3 ± 0.07	2 ± 0.07	3 ± 0.14

**Fig 3:**
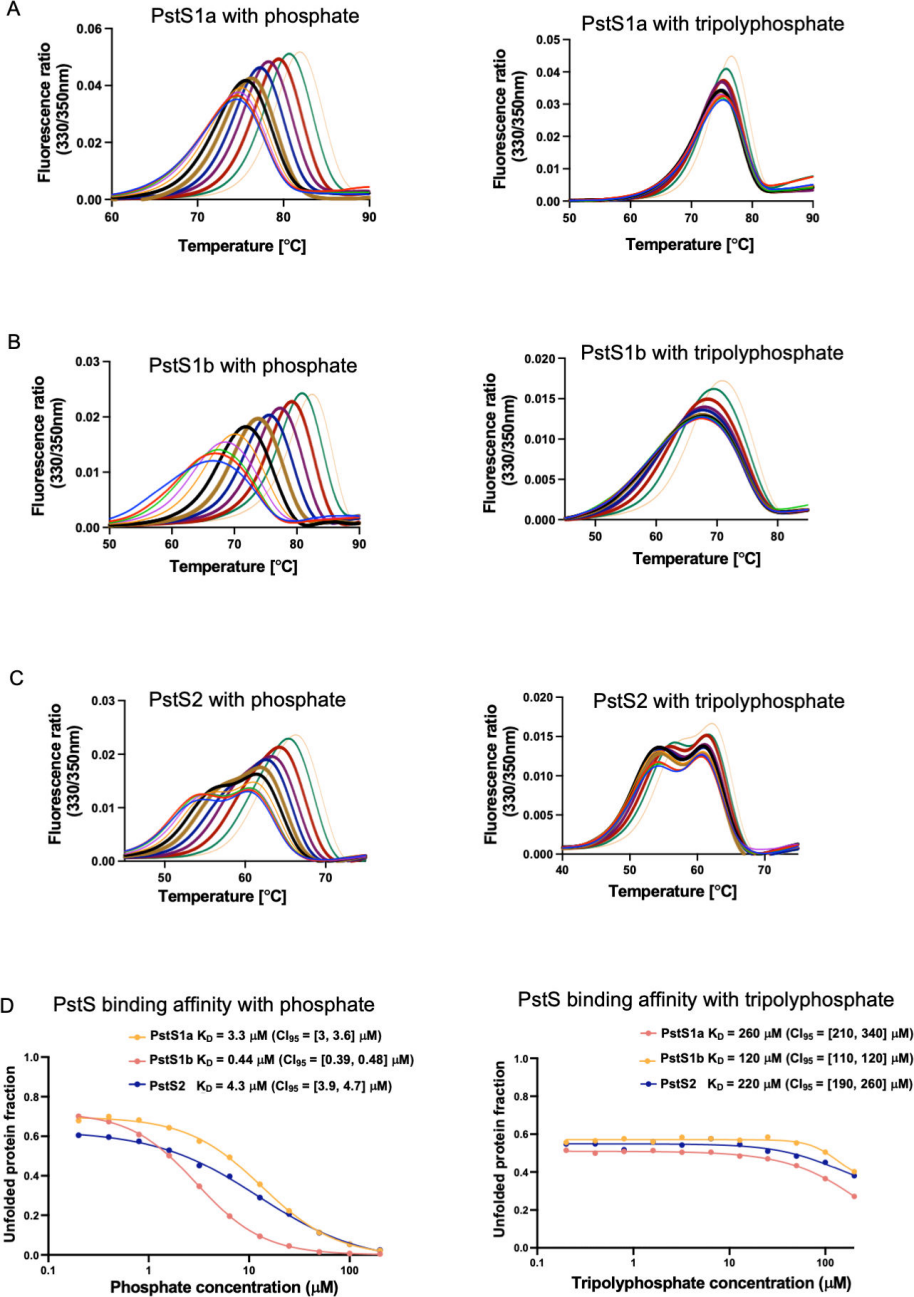
Thermal shift assay and binding affinity measurement of PstS proteins. DSF thermal melt assay for (**A**) PstS1a, (**B**) PstS1b, and (**C**) PstS2 with increasing concentration of phosphate and sodium tripolyphosphate and (**D**) binding affinity of PstS proteins calculated based on isothermal analysis of the DSF data (39, 40). The confidence interval [marginal asymmetric confidence interval at 95% confidence level (CI)] was estimated as suggested by Paketuryte et al. (41).

A fluorometric isothermal approach was used to determine the binding dissociation constants (*K*_D_) of all three PstS proteins in the presence of phosphate and sodium tripolyphosphate (39, 40). This method measures incremental shifts in melting temperature (i.e., increased thermal stability of the protein) in the presence of ligand, which is related to the proportion of folded versus unfolded protein at a single temperature (39, 40). This kinetic assay showed marked differences in ligand binding affinities in the presence of phosphate and polyphosphate, with PstS1b displaying the highest affinity in both cases. The *K*_D_ values for all PstS proteins in the presence of phosphate were found to be in the low micromolar range (0.44–4.3 µM), while the *K*_D_ values in the presence of tripolyphosphate were in the high micromolar range (120–260 μM) (Fig. 3D). It is unlikely that marine *Synechococcus* WH8102 isolated from an oligotrophic environment will encounter such high concentrations of tripolyphosphate, suggesting it to be a potentially non-cognate ligand for PstS proteins. Additionally, the binding affinity for PstS1b in the presence of phosphate was found to be 0.44 µM, almost 10-fold higher than PstS1a (*K*_D_ = 3.3 µM) and PstS2 (*K*_D_ = 4.3 µM).

### A threonine residue in the binding pocket of PstS1b is likely key for its higher phosphate affinity

**Fig 4:**
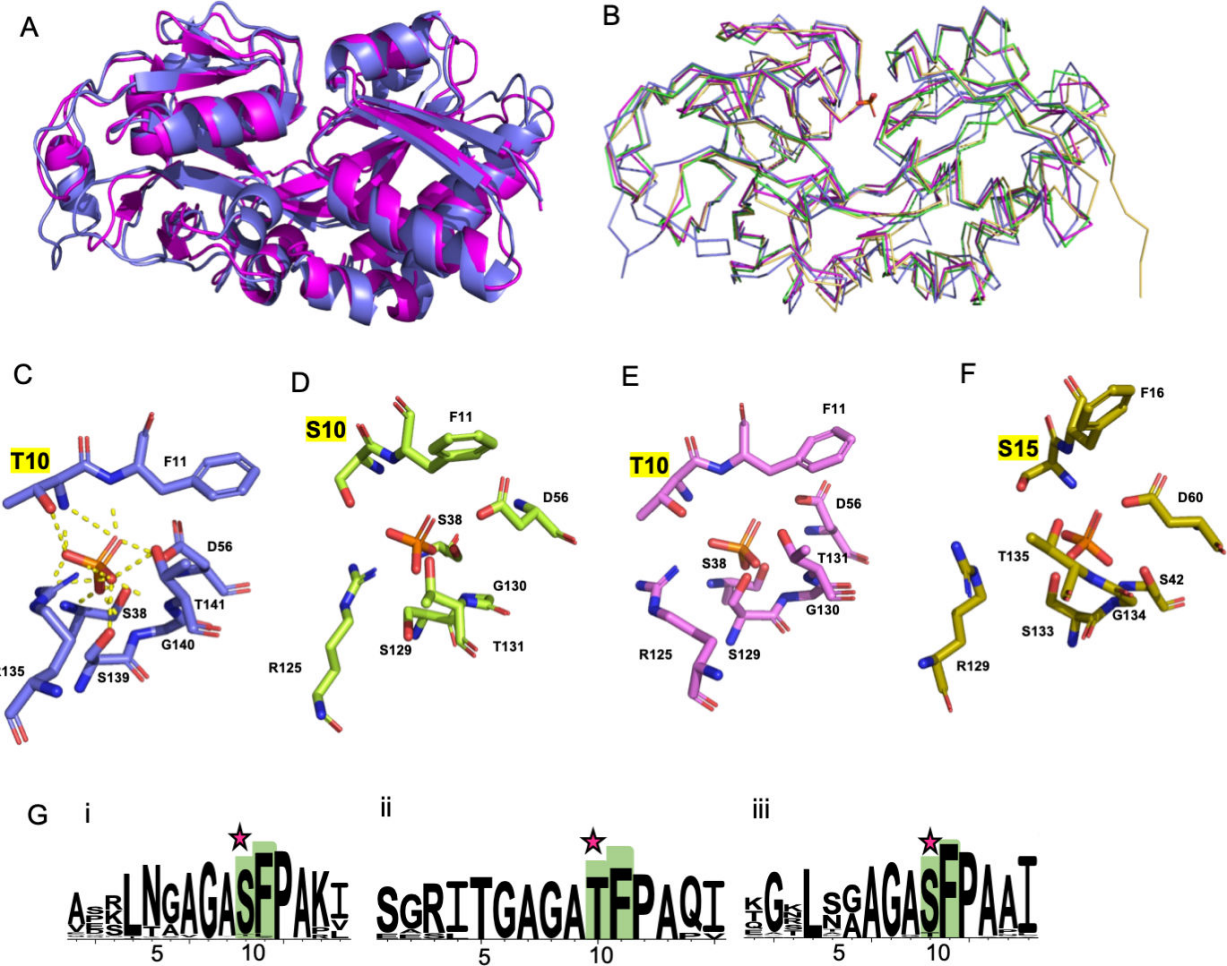
Comparison of the predicted structures of WH8102 PstS1a, PstS1b, and PstS2 with the *E. coli* PstS crystal structure. (**A**) An overlay of WH8102 PstS1b (pink) with the *E. coli* PstS crystal structure (PDB: 1ixH; marine blue) is shown as cartoon representatives. (**B**) An overlay of ribbon structures of *E. coli* PstS (marine blue) with WH8102 PstS1a (RMSD 1.274 Å, pink), PstS1b (RMSD 1.373 Å, green), and PstS2 (RMSD 1.479 Å, yellow). Details of the ligand binding residues for (**C**) *E. coli*, (**D**) PstS1a, (**E**) PstS1b, and (**F**) PstS2, with phosphate overlaid from the *E. coli* crystal structure showing the T10 residue of the *E. coli* crystal structure conserved in PstS1b but replaced with a serine residue at position S10 and S15 in Pst1a and PstS2, highlighted in yellow. (**G**) Sequence logo representation of the amino acid multiple sequence alignment of (i) PstS1a, (ii) PstS1b, and (iii) PstS2 proteins in the region of *E. coli* PstS T10 generated using WebLogo 3.7.12 (43). Binding residues are numbered according to amino acid residue per *E. coli* PstS. Binding residues involved in hydrogen bonding with phosphate are highlighted in green (the rest of the alignment is included as Fig. S3). The star above the amino acid alignments shows the T residue corresponding to T10 in *E. coli* PstS and its replacement as S in PstS1a and PstS2.

It was intriguing to observe that the two PstS1 proteins (PstS1a and PstS1b) share 73% amino acid sequence identity (Table S1), yet one exhibited approximately 10-fold higher affinity to phosphate. Therefore, we investigated the predicted active site phosphate binding residues of the PstS homologs. We compared the predicted structures of the WH8102 PstS homologs with the crystal structure of PstS from *E. coli* (PDB: 1ixH) (42). Predicted models for WH8102 PstS homologs showed a high degree of structural conservation with the crystal structure of *E. coli* PstS, with a root-mean-square deviation (RMSD) of <1.5°A between the Cα atoms of the protein backbone (Fig. 4B). The crystal structure of *E. coli* PstS shows phosphate held by 14 hydrogen bonds (H-bonds) from eight residues (T10, F11, S38, D56, R135, S139, G140, and T141). Three phosphate oxygen atoms (O1, O3, and O4) are involved in three H-bonds each, while five H-bonds stabilize O_2_ and contribute to the high affinity and specificity of PstS for phosphate (42). All of the eight binding residues in the *E. coli* active site are highly conserved in all homologs of WH8102 PstS except for *E. coli* threonine 10, which is conserved in PstS1b (Fig. 4E) but is replaced with serine in PstS1a and PstS2 (Fig. 4D and F).

Further investigation showed that PstS1b, which has approximately 10-fold higher affinity for phosphate and has the T10 residue, is primarily confined to *Synechococcus* clade III and also clusters separately from clade III PstS1a as shown in the phylogenetic tree (Fig. 1). Clade III *Synechococcus* are usually predominant in warm oligotrophic open oceanic regions (44). Hence, a very high-affinity phosphate binding protein most likely provides them with an additional competitive advantage to survive in ultraoligotrophic environments. Other freshwater and marine *Synechococcus* clades also have multiple PstS homologs present, but based on the conservation of the residue equivalent to *E. coli* T10, they may have binding affinities similar to PstS1a and PstS2 (Fig. 4G; Fig. S3).

One exception to the above generalization is observed in *Synechococcus* clade IV, which has only two strains (BL107 and CC902) sequenced to date, and both encode a PstS1b homolog. Clade IV is generally found in the coastal boundary zone alongside fluctuating phosphate concentrations (0.2–1.2 μM) (44). Studies have also suggested a “specialist” lifestyle for clade IV strains (12). Therefore, these strains may have evolved to encode a high-affinity SBP for phosphate uptake to survive and compete when encountering transient low-P conditions, such as stratified conditions during summer or when the water is more oligotrophic during the winter seasons due to off-shore water flow into coastal regions (45). It is also only during these conditions that clade III is also found to occur, albeit in low numbers, in coastal environments (45).

### The occurrence of *Synechococcus* PstS1b is restricted mainly to ultraoligotrophic marine regions

**Fig 5:**
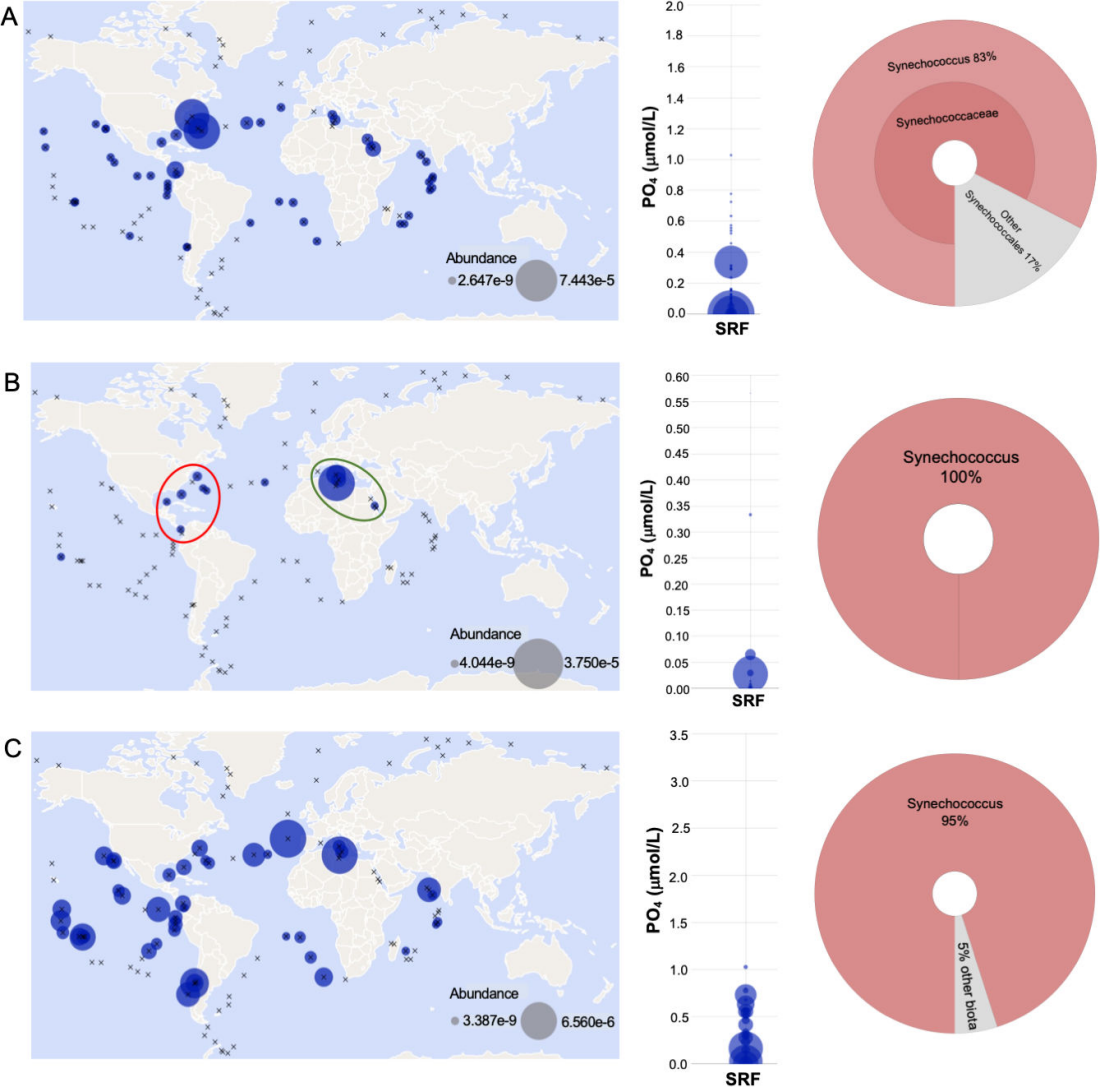
Ocean metatranscript distribution of *Synechococcus* WH8102 PstS homologs. Environmental abundance of (**A**) PstS1a, (**B**) PstS1b, and (**C**) PstS2 homologs extracted from the Tara Oceans MetaT data set (46, 47). Transcript abundance (expression data) is plotted for surface waters. The blue-filled circle size denotes measured abundance at a particular sampling site. Sampling sites are represented by “X.” The red circle highlights NAO stations (TARA 141-151), and the green circle highlights MS stations (TARA 011-025). The corresponding bubble plot for identified transcripts across surface water sampling depth (SRF) is shown as a function of phosphate concentration. The Krona plot shows the taxonomic distribution of PstS homolog hits selected to analyze meta transcriptome abundance.

We analyzed the geographical distribution and gene expression profiles of *Synechococcus pstS* homologs in oceanic regions worldwide to investigate whether the presence of *Synechococcus* PstS1b, the homolog with the highest affinity for phosphate, confers a competitive advantage in P-depleted regions (Fig. 5; Fig. S4). We utilized the metagenomes and metatranscriptomes available from the Ocean Microbial Reference Gene Catalogue (OM-RGC) for this analysis (46, 47). Our analysis revealed that *Synechococcus* PstS1a and PstS2 genes or transcripts are widely distributed across different oceanic regions, indicating their presence in diverse ecosystems. In contrast, the distribution of *Synechococcus* Pst1b is primarily restricted to ultraoligotrophic oceanic regions, specifically the North Atlantic Ocean (NAO, stations TARA_141-TARA_151) and the Mediterranean Sea (MS, stations TARA_011-TARA_025). Further examination of the data showed that *Synechococcus* PstS1a and PstS2 are found across a wide range of phosphorus concentrations. They are observed in the surface water layer (SRF) with phosphorus concentrations ranging from 0.01 to 1 µmol/L. In contrast, PstS1b is predominantly present in oceanic regions with low phosphorus concentrations (<0.16 µmol/L). This is consistent with the relative phosphate binding affinities for these proteins from WH8102 (Fig. 3D).

Previous studies investigating marine cyanobacterial community dynamics in various oceanic regions (48–50) have demonstrated that *Synechococcus* clade III strains, such as WH8102, do not show a latitudinal preference. Instead, its distribution is confined to a narrow range of macronutrient availability (44). This restricted distribution of *Synechococcus* WH8102 PstS1b to subtropical ultraoligotrophic oceanic niches aligns with our findings, indicating that the presence of the high-affinity binding protein PstS1b primarily confined to clade III likely provides an additional competitive advantage for the survival of marine picocyanobacteria such as *Synechococcus* sp. WH8102, in P-deplete environments.

### Conclusions

Our genomic context analysis and protein biophysical data suggest distinct ecological roles for WH8102 PstS homologs. The PstS1b protein shows the highest affinity for phosphate which is typically present in low nanomolar ranges in oligotrophic oceans. The genomic location of *pstS1b* adjacent to stress response genes suggests its involvement in survival during stressful conditions in low-P environments and potentially aids in scavenging inorganic phosphate. On the other hand, PstS1a, with a 10-fold lower affinity for phosphate than PstS1b, likely facilitates the uptake of recycled phosphate resulting from organic phosphate cleavage, as this gene is co-transcribed with the *ptrA* transcriptional regulatory gene that controls expression of the alkaline phosphatase genes. Although the role of PstS2 is not fully understood, it is possible that it is responsible for inorganic phosphate acquisition when environmental phosphate concentrations are transiently higher due to events such as upwelling, coastal runoff, or viral lysis.

Our proposed P uptake strategy for WH8102 aligns with previous studies in various bacteria demonstrating the benefits of having multiple transporters with varying affinities and transport rates for the same nutrient. This strategy may allow cells to finely regulate their nutrient uptake based on the availability and concentration of the substrate in the environment. This balance helps address the “rate-affinity trade-off,” where high-affinity SBPs are more effective at scavenging nutrients at low concentrations, while low-affinity SBPs are more efficient for rapid substrate turnover (51). The P stress response potentially provides WH8102 with a flexible and adaptive nutrient acquisition system, allowing it to fine tune its phosphate uptake strategy according to the prevailing environmental conditions. By dynamically adjusting its utilization of high- and low-affinity P transporters, WH8102 likely maximizes its ability to acquire P while minimizing energy expenditure.

## MATERIALS AND METHODS

### Phylogenetic analyses

The *pstS* sequences belonging to gene clusters CK_00043821 (*pstS)* and CK_00000023 (*pstS*2) containing 73 and 51 orthologous *Synechococcus/Cyanobium* sequences, respectively, were extracted from the Cyanorak database (www.sb-roscoff.fr/cyanorak) (27). Phylogenetic analyses of these sequences were conducted using a modified method given by Wilding et al. (52). Briefly, all *Synechococcus* PstS homolog sequences were aligned using the L-INS-I option of MAFFT, and the phylogenetic tree was inferred using IQ-Tree (29). The optimal model using -TESTONLY option was found to be WAG + G4. A final phylogenetic tree was generated using the inferred maximum likelihood model and visualized using iTOL (30).

### Recombinant protein expression and purification

The protein sequences of SYNW01018 (PstS1a), SYNW01815 (PstS1b), and SYNW02507 (PstS2) were obtained from the Cyanorak database (27) and were analyzed using SignalP5.0 server (53). The N-terminal truncated target genes (Table S2) were PCR amplified from WH8102 gDNA, incorporating vector-specific overhang regions for heterologous expression in *E. coli*. Ligation-independent cloning (Clontech) was carried out using *Kpn*I and *Hind*III restriction sites to incorporate an N-terminal hexahistidine tag (His-tag) with a 3C protease cleavage site (54, 55) into pOPIN-F (for PstS1a and PstS2) and pOPIN-S (for PstS1b) vector. All cloned plasmids were transformed into *E. coli* Lemo21(DE3) cells and expressed to high density using the auto-induction method (56). The IMAC technique, as described previously (57), was used to purify all target proteins, which were then desalted using SEC with a Superdex HiLoad 200 16/600 column (GE Healthcare) pre-equilibrated in a gel filtration buffer [HEPES (20 mM, pH 7.5), NaCl (200 mM), TCEP (1 mM)]. The His-tag from pooled protein fractions was then cleaved using 3C protease. De-tagged protein was collected using Reverse IMAC using a prepacked Ni-NTA column (1 mL, GE Healthcare) pre-equilibrated in gel filtration buffer. Protein-containing fractions were concentrated using centrifugation (10-kDa MWCO, Vivaspin6, 2,000 × *g*, 4°C) and snap frozen (50-µL aliquots) in liquid N_2_. The purity of the recovered protein sample was verified using SDS-PAGE, showing a single band when visualized with Coomassie blue dye (Fig. S1).

The evaluation of all PstS proteins’ native mass in solution was carried out using analytical SEC procedures on a Superdex 200 10/300 GL column (GE-Healthcare) equilibrated in a gel filtration buffer. Elution times were calibrated using a protein standard mix (15–600 kDa; Sigma-Aldrich), and the void volume (*V*_0_) was estimated using blue dextran. Partition coefficients (*K*_av_) were calculated from elution volumes and used to derive a plot of *K*_av_ against log(*M*_R_) to allow the interpolation of unknown masses based on elution volume. The line of best fit was given as log(*M*_R_) *=* −3(*K*_av_) + 5.9214, with a correlation coefficient (*R*^2^) of 0.9972.

### Substrate specificity and binding affinity determination of PstS proteins

Substrate specificity of PstS proteins was tested against different organic and inorganic phosphorus sources, including potassium phosphate, sodium phosphite, sodium β-glycerophosphate, and sodium tripolyphosphate using nano DSF. Nano DSF is an intrinsic fluorescence high-throughput method to study thermal shifts caused by protein-ligand binding. It monitors protein unfolding as a function of temperature and measures the intensity ratio of fluorescence at 350 nm/330 nm based on the intrinsic tryptophan fluorescence (58). A range of protein concentrations was tested to determine the optimal signal-to-noise ratio, requiring a low protein concentration (10 µM). To determine the potential ligand, each of the purified PstS proteins (10 µM) was mixed with phosphate, phosphite, β-glycerophosphate, and sodium tripolyphosphate (200 µM each). Protein and ligand mixtures were transferred into standard grade capillaries (Nanotemper) and heated over a temperature gradient of 20°C–95°C at a ramp rate of 1 °C min^−1^ using a Prometheus NT.48 fluorimeter (Nanotemper). Excitation power was pre-adjusted to obtain fluorescence readings >2,000 relative fluorescence units at 330 nm (F330) and 350 nm (F350). Thermal melt curves were analyzed using the same software (PR.ThermControl).

The binding affinity for potential ligands determined from the initial substrate screening was calculated using a modified DSF assay (40). Briefly, each of the PstS proteins (10 µM) was mixed with increasing concentrations (0.2–200 µM) of phosphate and tripolyphosphate. Samples were heated over a temperature gradient of 20°C–95°C at a ramp rate of 1 °C min^−1^ with a Prometheus NT.48 fluorimeter. Data were processed using a web-based Fold Affinity tool (39), and binding constant values were determined. The confidence interval (marginal asymmetric confidence interval at 95% confidence level) was estimated as suggested by Paketuryte et al. (41). Processed results were plotted using GraphPad Prism.

### *Synechococcus* sp. WH8102 PstS protein structure prediction and analysis

The structure of PstS proteins from WH8102 was predicted using RoseTTAFold through an online interface Robetta (59). SignalP 5.0 (53) truncated query sequences of PstS1a, PstS1b, and PstS2 were uploaded into the interface, and structure prediction was performed using default parameters. The top-ranked model for each of the three proteins was then analyzed further by comparing it to *E. coli* PstS (PDB id: 1ixH) to identify active site binding residues using PyMOL 2.5.2. To highlight the conservation of binding residues in PstS proteins from all marine *Synechococcus* available in the Cyanorak database along with freshwater *Synechocystis* sp. 6803 and *E. coli*, respective amino acid sequences were aligned using MAFFT (28). Sequence representations of alignment were generated using WebLogo 3.7.12 (43). The RosettaFold predicted structures for the three PstS proteins were also compared with AlphaFold2 predictions from the UniProt database, showing strong conservation (RMSD 0.95 Å–1.16 Å) between the predicted structure pairs from the two methods.

### Biogeography of PstS proteins

Biogeography and environmental context of PstS proteins of interest belonging to WH8102 were analyzed using Ocean Gene Atlas to visualize data from the *Tara* Oceans expedition (46, 47). The protein sequence for each of the targets was used to search the Ocean Gene Atlas against the “OM-RGCv2 + T” and “OM-RGC-v2 + G” data sets using default parameters. To ensure that only *Synechococcus* sequences were included and the homolog matches did not overlap, a high filter expectation value (e-value −185, −165, and −155 for PstS1a, PstS1b, and PstS2, respectively) was selected. The results were visualized using phosphate as an environmental parameter of interest.

## Data Availability

All study data are included in the article and/or supplemental files.

## SUPPLEMENTAL MATERIAL

Supplemental material spectrum.02786-23-s0001.pdf Figures S1 to S4; Tables S1 and S2.

ASM does not own the copyrights to Supplemental Material that may be linked to, or accessed through, an article. The authors have granted ASM a non-exclusive, world-wide license to publish the Supplemental Material files. Please contact the corresponding author directly for reuse.

## References

[1] Tetu SG, Brahamsha B, Johnson DA, Tai V, Phillippy K, Palenik B, Paulsen IT. 2009. Microarray analysis of phosphate regulation in the marine cyanobacterium Synechococcus sp. WH8102. ISME J 3:835–849. doi:10.1038/ismej.2009.31 19340084

[2] Ammerman JW, Hood RR, Case DA, Cotner JB. 2003. Phosphorus deficiency in the Atlantic: an emerging paradigm in oceanography. EoS Transactions 84:165–170. doi:10.1029/2003EO180001

[3] Karl DM, Tien G. 1997. Temporal variability in dissolved phosphorus concentrations in the subtropical North Pacific ocean. Mar Chem 56:77–96. doi:10.1016/S0304-4203(96)00081-3

[4] Cotner JB, Ammerman JW, Peele ER, Bentzen E. 1997. Phosphorus-limited bacterioplankton growth in the Sargasso sea. Aquat Microb Ecol 13:141–149. doi:10.3354/ame013141

[5] Scanlan DJ, Ostrowski M, Mazard S, Dufresne A, Garczarek L, Hess WR, Post AF, Hagemann M, Paulsen I, Partensky F. 2009. Ecological genomics of marine picocyanobacteria. Microbiol Mol Biol Rev 73:249–299. doi:10.1128/MMBR.00035-08 19487728 PMC2698417

[6] Partensky F, Hess WR, Vaulot D. 1999. Prochlorococcus, a marine photosynthetic prokaryote of global significance . Microbiol Mol Biol Rev 63:106–127. doi:10.1128/MMBR.63.1.106-127.1999 10066832 PMC98958

[7] Moore LR, Ostrowski M, Scanlan DJ, Feren K, Sweetsir T. 2005. Ecotypic variation in phosphorus-acquisition mechanisms within marine picocyanobacteria. Aquat Microb Ecol 39:257–269. doi:10.3354/ame039257

[8] Martiny AC, Coleman ML, Chisholm SW. 2006. Phosphate acquisition genes in Prochlorococcus ecotypes: evidence for genome-wide adaptation. Proc Natl Acad Sci U S A 103:12552–12557. doi:10.1073/pnas.0601301103 16895994 PMC1567916

[9] Fu FX, Zhang Y, Feng Y, Hutchins DA. 2006. Phosphate and ATP uptake and growth kinetics in axenic cultures of the cyanobacterium Synechococcus CCMP 1334. Eur J Phycol 41:15–28. doi:10.1080/09670260500505037

[10] Van Mooy BAS, Fredricks HF, Pedler BE, Dyhrman ST, Karl DM, Koblízek M, Lomas MW, Mincer TJ, Moore LR, Moutin T, Rappé MS, Webb EA. 2009. Phytoplankton in the ocean use non-phosphorus lipids in response to phosphorus scarcity. Nature 458:69–72. doi:10.1038/nature07659 19182781

[11] Palenik B, Brahamsha B, Larimer FW, Land M, Hauser L, Chain P, Lamerdin J, Regala W, Allen EE, McCarren J, Paulsen I, Dufresne A, Partensky F, Webb EA, Waterbury J. 2003. The genome of a motile marine Synechococcus. Nature 424:1037–1042. doi:10.1038/nature01943 12917641

[12] Dufresne A, Ostrowski M, Scanlan DJ, Garczarek L, Mazard S, Palenik BP, Paulsen IT, de Marsac NT, Wincker P, Dossat C, Ferriera S, Johnson J, Post AF, Hess WR, Partensky F. 2008. Unraveling the genomic mosaic of a ubiquitous genus of marine cyanobacteria. Genome Biol 9:1–16. doi:10.1186/gb-2008-9-5-r90 PMC2441476 18507822

[13] Venter JC, Remington K, Heidelberg JF, Halpern AL, Rusch D, Eisen JA, Wu D, Paulsen I, Nelson KE, Nelson W, Fouts DE, Levy S, Knap AH, Lomas MW, Nealson K, White O, Peterson J, Hoffman J, Parsons R, Baden-Tillson H, Pfannkoch C, Rogers Y-H, Smith HO. 2004. Environmental genome shotgun sequencing of the Sargasso sea. Science 304:66–74. doi:10.1126/science.1093857 15001713

[14] Scanlan DJ, Mann NH, Carr NG. 1993. The response of the picoplanktonic marine cyanobacterium Synechococcus species WH7803 to phosphate starvation involves a protein homologous to the periplasmic phosphate‐binding protein of Escherichia coli. Mol Microbiol 10:181–191. doi:10.1111/j.1365-2958.1993.tb00914.x 7968514

[15] Scanlan DJ, Silman NJ, Donald KM, Wilson WH, Carr NG, Joint I, Mann NH. 1997. An immunological approach to detect phosphate stress in populations and single cells of photosynthetic picoplankton. Appl Environ Microbiol 63:2411–2420. doi:10.1128/aem.63.6.2411-2420.1997 9172363 PMC168535

[16] Nagaya M, Aiba H, Mizuno T. 1994. The sphR product, a two-component system response regulator protein, regulates phosphate assimilation in Synechococcus sp. strain PCC 7942 by binding to two sites upstream from the phoA promoter. J Bacteriol 176:2210–2215. doi:10.1128/jb.176.8.2210-2215.1994 8157591 PMC205341

[17] Suzuki S, Ferjani A, Suzuki I, Murata N. 2004. The SphS-SphR two component system is the exclusive sensor for the induction of gene expression in response to phosphate limitation in Synechocystis. J Biol Chem 279:13234–13240. doi:10.1074/jbc.M313358200 14707128

[18] Pitt FD, Mazard S, Humphreys L, Scanlan DJ. 2010. Functional characterization of Synechocystis sp. strain PCC 6803 pst1 and pst2 gene clusters reveals a novel strategy for phosphate uptake in a freshwater cyanobacterium. J Bacteriol 192:3512–3523. doi:10.1128/JB.00258-10 20435726 PMC2897655

[19] Aiba H, Mizuno T. 1994. A novel gene whose expression is regulated by the response‐regulator, SphR, in response to phosphate limitation in Synechococcus species PCC7942. Mol Microbiol 13:25–34. doi:10.1111/j.1365-2958.1994.tb00399.x 7741855

[20] Mann NH, Scanlan DJ. 1994. The SphX protein of Synechococcus species PCC 7942 belongs to a family of phosphate-binding proteins. Mol Microbiol 14:595–596. doi:10.1111/j.1365-2958.1994.tb02192.x 7885237

[21] Davidson AL, Dassa E, Orelle C, Chen J. 2008. Structure, function, and evolution of bacterial ATP-binding cassette systems. Microbiol Mol Biol Rev 72:317–364, doi:10.1128/MMBR.00031-07 18535149 PMC2415747

[22] Tam R, Saier MH. 1993. Structural, functional, and evolutionary relationships among extracellular solute-binding receptors of bacteria. Microbiol Rev 57:320–346. doi:10.1128/mr.57.2.320-346.1993 8336670 PMC372912

[23] Thomas GH. 2010. Homes for the orphans: utilization of multiple substrate-binding proteins by ABC transporters: microcommentary. Mol Microbiol 75:6–9. doi:10.1111/j.1365-2958.2009.06961.x 19919676

[24] Chen C, Malek AA, Wargo MJ, Hogan DA, Beattie GA. 2010. The ATP-binding cassette transporter Cbc (choline/betaine/carnitine) recruits multiple substrate-binding proteins with strong specificity for distinct quaternary ammonium compounds. Mol Microbiol 75:29–45. doi:10.1111/j.1365-2958.2009.06962.x 19919675 PMC5503199

[25] Nikaido K, Ames GFL. 1999. One intact ATP-binding subunit is sufficient to support ATP hydrolysis and translocation in an ABC transporter, the histidine permease. J Biol Chem 274:26727–26735. doi:10.1074/jbc.274.38.26727 10480876

[26] Waterbury J, Watson S, Valois F, Franks D. 1986. Biological and ecological characterization of the marine unicellular cyanobacterium. Can Bull Fish Aquat Sci 214:71–120.

[27] Garczarek L, Guyet U, Doré H, Farrant GK, Hoebeke M, Brillet-Guéguen L, Bisch A, Ferrieux M, Siltanen J, Corre E, Le Corguillé G, Ratin M, Pitt FD, Ostrowski M, Conan M, Siegel A, Labadie K, Aury J-M, Wincker P, Scanlan DJ, Partensky F. 2021. Cyanorak v2.1: a scalable information system dedicated to the visualization and expert curation of marine and brackish picocyanobacteria genomes. Nucleic Acids Res 49:D667–D676. doi:10.1093/nar/gkaa958 33125079 PMC7779031

[28] Katoh K, Standley DM. 2013. MAFFT multiple sequence alignment software version 7: improvements in performance and usability. Mol Biol Evol 30:772–780. doi:10.1093/molbev/mst010 23329690 PMC3603318

[29] Nguyen L-T, Schmidt HA, von Haeseler A, Minh BQ. 2015. IQ-TREE: a fast and effective stochastic algorithm for estimating maximum-likelihood phylogenies. Mol Biol Evol 32:268–274. doi:10.1093/molbev/msu300 25371430 PMC4271533

[30] Letunic I, Bork P. 2019. Interactive tree of life (iTOL) V4: recent updates and new developments. Nucleic Acids Res 47:W256–W259. doi:10.1093/nar/gkz239 30931475 PMC6602468

[31] Lynch M, Conery JS. 2000. The evolutionary fate and consequences of duplicate genes. Science 290:1151–1155. doi:10.1126/science.290.5494.1151 11073452

[32] Sanchez-Perez G, Mira A, Nyiro G, Pasić L, Rodriguez-Valera F. 2008. Adapting to environmental changes using specialized paralogs. Trends Genet 24:154–158. doi:10.1016/j.tig.2008.01.002 18325625

[33] Ostrowski M, Mazard S, Tetu SG, Phillippy K, Johnson A, Palenik B, Paulsen IT, Scanlan DJ. 2010. Ptra is required for coordinate regulation of gene expression during phosphate stress in a marine Synechococcus. ISME J 4:908–921. doi:10.1038/ismej.2010.24 20376102

[34] Gilchrist CLM, Chooi YH. 2021. Clinker & clustermap.js: automatic generation of gene cluster comparison figures. Bioinformatics 37:2473–2475. doi:10.1093/bioinformatics/btab007 33459763

[35] Enomoto G, Wallner T, Wilde A. 2023. Control of light-dependent behaviour in cyanobacteria by the second messenger cyclic Di-GMP. Microlife 4:uqad019. doi:10.1093/femsml/uqad019 37223735 PMC10124867

[36] Kammerscheit X, Chauvat F, Cassier-Chauvat C. 2019. From cyanobacteria to human, MAPEG-type glutathione-S-transferases operate in cell tolerance to heat, cold, and lipid peroxidation. Front Microbiol 10:2248. doi:10.3389/fmicb.2019.02248 31681188 PMC6798054

[37] Chamnongpol S, Groisman EA. 2000. Acetyl phosphate-dependent activation of a mutant PhoP response regulator that functions independently of its cognate sensor kinase. J Mol Biol 300:291–305. doi:10.1006/jmbi.2000.3848 10873466

[38] Tetu SG, Johnson DA, Varkey D, Phillippy K, Stuart RK, Dupont CL, Hassan KA, Palenik B, Paulsen IT. 2013. Impact of DNA damaging agents on genome-wide transcriptional profiles in two marine Synechococcus species. Front Microbiol 4:232. doi:10.3389/fmicb.2013.00232 23966990 PMC3744912

[39] Niebling S, Burastero O, Bürgi J, Günther C, Defelipe LA, Sander S, Gattkowski E, Anjanappa R, Wilmanns M, Springer S, Tidow H, García-Alai M. 2021. FoldAffinity: binding affinities from nDSF experiments. Sci Rep 11:9572. doi:10.1038/s41598-021-88985-z 33953265 PMC8099913

[40] Bai N, Roder H, Dickson A, Karanicolas J. 2019. Isothermal analysis of ThermoFluor data can readily provide quantitative binding affinities. Sci Rep 9:2650. doi:10.1038/s41598-018-37072-x 30804351 PMC6389909

[41] Paketurytė V, Petrauskas V, Zubrienė A, Abian O, Bastos M, Chen W-Y, Moreno MJ, Krainer G, Linkuvienė V, Sedivy A, Velazquez-Campoy A, Williams MA, Matulis D. 2021. Uncertainty in protein–ligand binding constants: asymmetric confidence intervals versus standard errors. Eur Biophys J 50:661–670. doi:10.1007/s00249-021-01518-4 33837826

[42] Luecke H, Quiocho FA. 1990. High specificity of a phosphate transport protein determined by hydrogen bonds. Nature 347:402–406. doi:10.1038/347402a0 2215649

[43] Crooks GE, Hon G, Chandonia JM, Brenner SE. 2004. WebLogo: a sequence logo generator. Genome Res 14:1188–1190. doi:10.1101/gr.849004 15173120 PMC419797

[44] Zwirglmaier K, Jardillier L, Ostrowski M, Mazard S, Garczarek L, Vaulot D, Not F, Massana R, Ulloa O, Scanlan DJ. 2008. Global phylogeography of marine Synechococcus and Prochlorococcus reveals a distinct partitioning of lineages among oceanic biomes. Environ Microbiol 10:147–161. doi:10.1111/j.1462-2920.2007.01440.x 17900271

[45] Tai V, Palenik B. 2009. Temporal variation of Synechococcus clades at a coastal Pacific ocean monitoring site. ISME J 3:903–915. doi:10.1038/ismej.2009.35 19360028

[46] Villar E, Vannier T, Vernette C, Lescot M, Cuenca M, Alexandre A, Bachelerie P, Rosnet T, Pelletier E, Sunagawa S, Hingamp P. 2018. The ocean gene Atlas: exploring the biogeography of plankton genes online. Nucleic Acids Res 46:W289–W295. doi:10.1093/nar/gky376 29788376 PMC6030836

[47] Sunagawa S, Coelho LP, Chaffron S, Kultima JR, Labadie K, Salazar G, Djahanschiri B, Zeller G, Mende DR, Alberti A, et al.. 2015. Structure and function of the global ocean microbiome. Science 348:1261359. doi:10.1126/science.1261359 25999513

[48] Fuller Nicholas J., West NJ, Marie D, Yallop M, Rivlin T, Post AF, Scanlan DJ. 2005. Dynamics of community structure and phosphate status of picocyanobacterial populations in the Gulf of Aqaba, red sea. Limnol. Oceanogr 50:363–375. doi:10.4319/lo.2005.50.1.0363

[49] Zwirglmaier K, Heywood JL, Chamberlain K, Woodward EMS, Zubkov MV, Scanlan DJ. 2007. Basin-scale distribution patterns of picocyanobacterial lineages in the Atlantic ocean. Environ Microbiol 9:1278–1290. doi:10.1111/j.1462-2920.2007.01246.x 17472640

[50] Fuller NJ, Tarran GA, Yallop M, Orcutt KM, Scanlan DJ. 2006. Molecular analysis of picocyanobacterial community structure along an Arabian sea transect reveals distinct spatial separation of lineages. Limnol Oceanograp 51:2515–2526. doi:10.4319/lo.2006.51.6.2515

[51] Montaño-Gutierrez LF, Correia K, Swain PS. 2022. Multiple nutrient transporters enable cells to mitigate a rate-affinity tradeoff. PLoS Comput Biol 18:e1010060. doi:10.1371/journal.pcbi.1010060 35468136 PMC9071158

[52] Wilding M, Peat TS, Kalyaanamoorthy S, Newman J, Scott C, Jermiin LS. 2017. Reverse engineering: transaminase biocatalyst development using ancestral sequence reconstruction. Green Chem 19:5375–5380. doi:10.1039/C7GC02343J

[53] Almagro Armenteros JJ, Tsirigos KD, Sønderby CK, Petersen TN, Winther O, Brunak S, von Heijne G, Nielsen H. 2019. Signalp 5.0 improves signal peptide predictions using deep neural networks. Nat Biotechnol 37:420–423. doi:10.1038/s41587-019-0036-z 30778233

[54] Aslanidis C, de Jong PJ. 1990. Ligation-independent cloning of PCR products (LIC-PCR). Nucleic Acids Res 18:6069–6074. doi:10.1093/nar/18.20.6069 2235490 PMC332407

[55] Berrow NS, Alderton D, Sainsbury S, Nettleship J, Assenberg R, Rahman N, Stuart DI, Owens RJ. 2007. A versatile ligation-independent cloning method suitable for high-throughput expression screening applications. Nucleic Acids Res 35:e45. doi:10.1093/nar/gkm047 17317681 PMC1874605

[56] Studier FW. 2005. Protein production by auto-induction in high density shaking cultures. Protein Expr Purif 41:207–234. doi:10.1016/j.pep.2005.01.016 15915565

[57] Ford BA, Ranjit P, Mabbutt BC, Paulsen IT, Shah BS. 2022. Prox from marine Synechococcus spp. show a sole preference for glycine-betaine with differential affinity between ecotypes. Environ Microbiol 24:6071–6085. doi:10.1111/1462-2920.16168 36054310 PMC10087775

[58] Chattopadhyay G, Varadarajan R. 2019. Facile measurement of protein stability and folding kinetics using a nano differential scanning fluorimeter. Protein Sci 28:1127–1134. doi:10.1002/pro.3622 30993730 PMC6511731

[59] Kim DE, Chivian D, Baker D. 2004. Protein structure prediction and analysis using the Robetta server. Nucleic Acids Res 32:W526–31. doi:10.1093/nar/gkh468 15215442 PMC441606

