# Supplementary material for: Substrate specificity and ecological significance of PstS homologs in phosphorus uptake in marine Synechococcus sp. WH8102
Source: Microbiol Spectr. 2024 Jan 5;12(2):e02786-23. doi: 10.1128/spectrum.02786-23 (PMC10846223; doi:10.1128/spectrum.02786-23)
Supplement: Supplemental material — Figures S1 to S4; Tables S1 and S2. [file spectrum.02786-23-s0001.pdf]

**Substrate specificity and ecological significance of PstS homologs in phosphorus uptake in marine *Synechococcus sp.* WH8102**

**Supplementary Figure S1: Analytical size exclusion traces of three PstS proteins.** Chromatograms of (A) PstS1a (B) PstS1b (C) PstS2 obtained from size exclusion chromatography (SEC). Each peak indicates collected protein eluate. Purified protein fractions analysed using SDS-PAGE are shown as inset for each purified protein. Void volume ($V_0$) is marked with an arrow. Analytical SEC was performed using Superdex 200 10/300 GL column (GE-Healthcare).

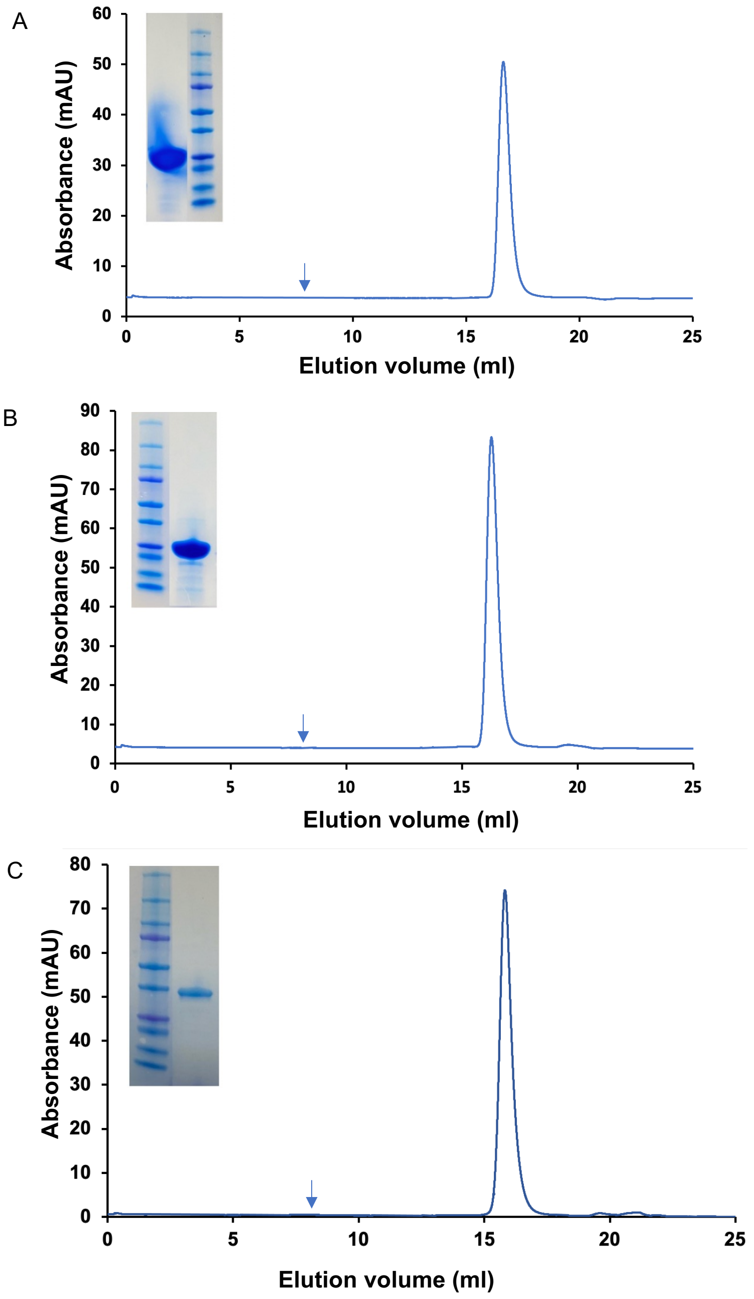

**Supplementary Figure S2: Thermal melt curves of PstS homologs**. Melt curves of (A) PstS1a, (B) PstS1b and (C) PstS2 in the presence of phosphate (red), phosphite (green), ß-glycerophosphate (pink), and sodium tripolyphosphate (maroon), showing a significant increase in melting temperature ($\Delta$ $T_M$) of protein in the presence of phosphate for all three proteins.

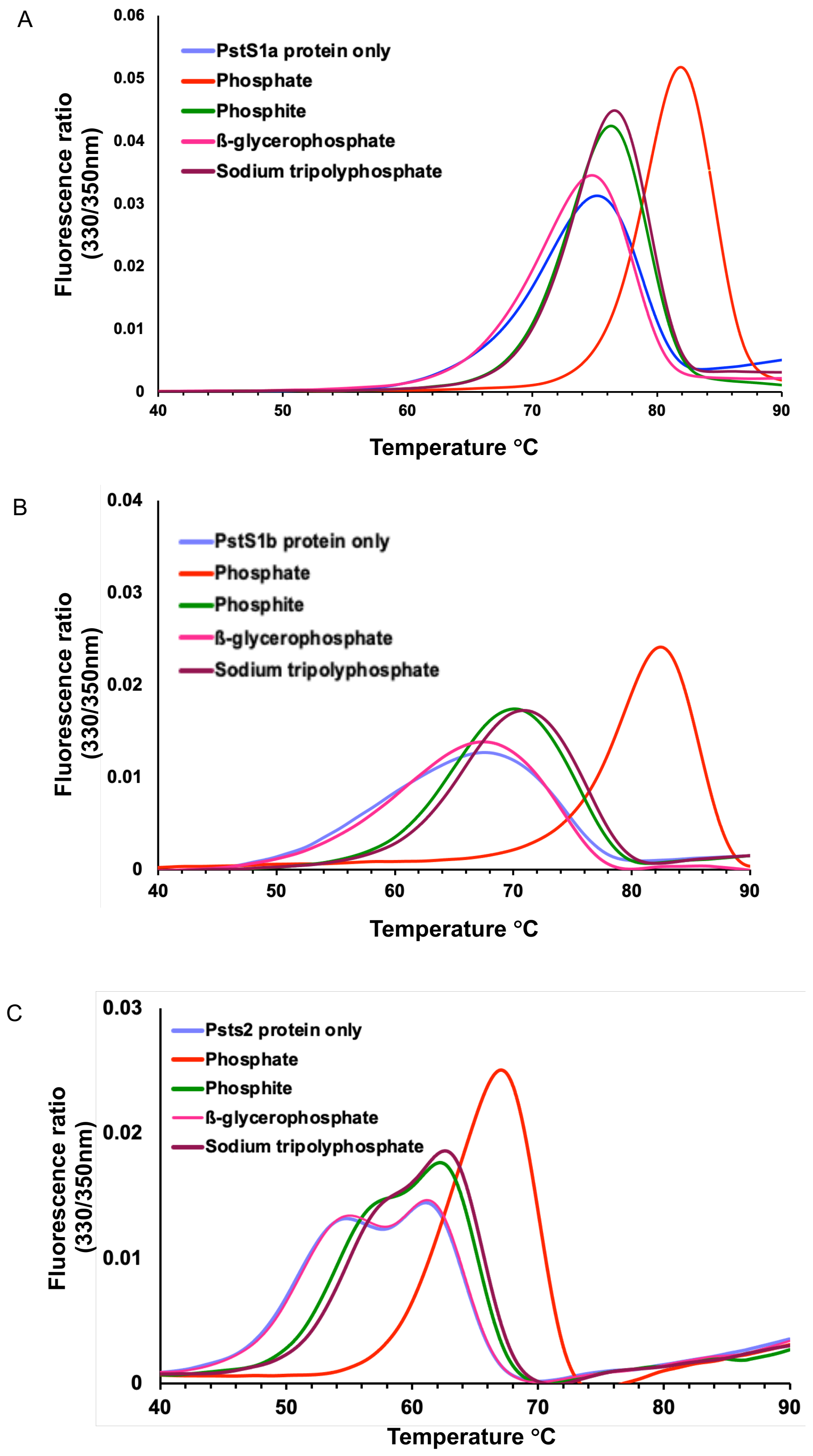

**Supplementary Figure S3: Sequence logo representation of *Synechococcus* PstS amino acid sequences.** Sequence logo representation of (A) PstS1a, (B) PstS1b and (C) PstS2 proteins were generated using WebLogo 3.7.12 (58). Binding residues involved in binding Pi are highlighted in green. A red star above an amino acid shows a threonine residue in the binding site of PstS1b and a serine residue for PstS1a and PstS2.

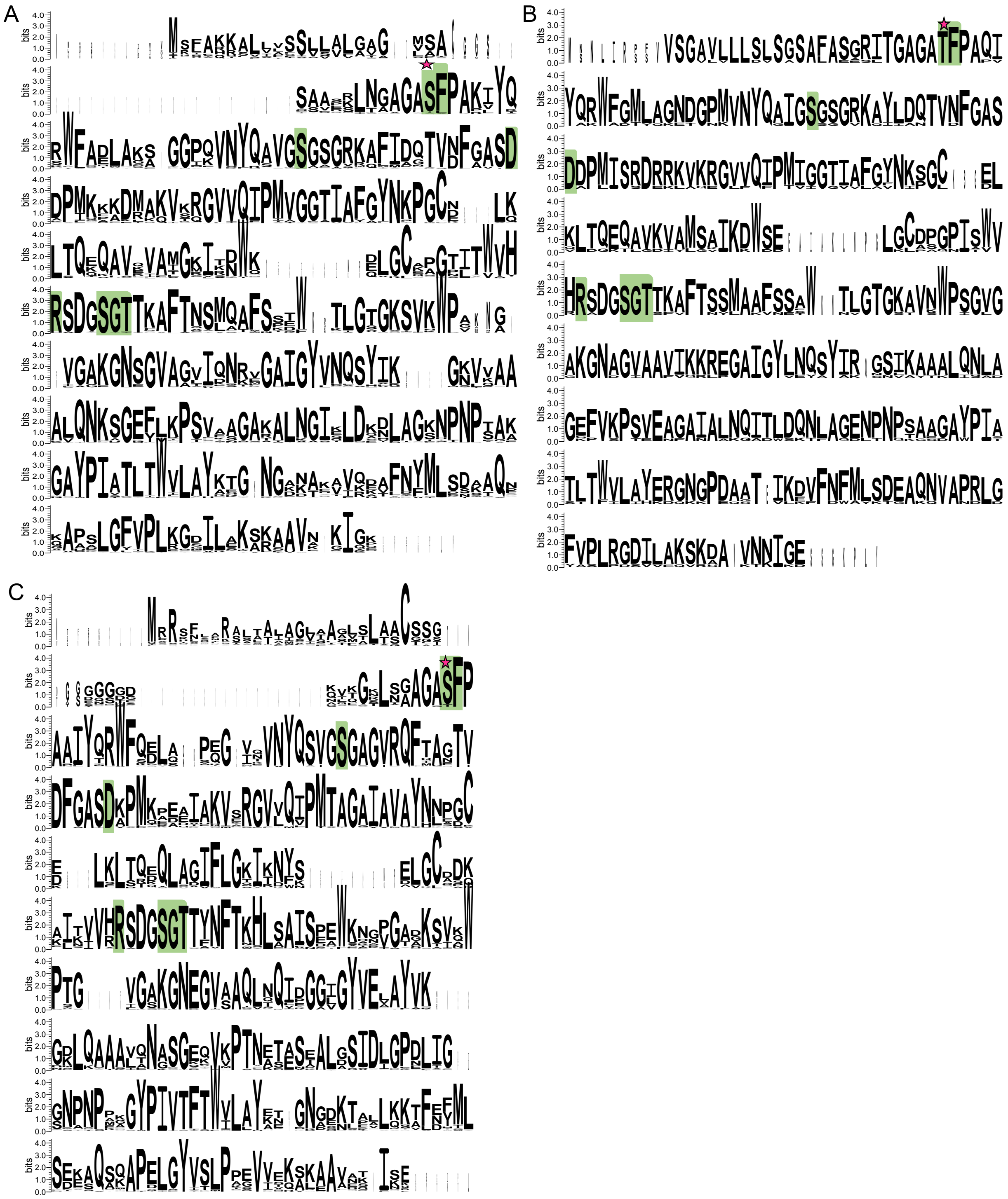

**Supplementary Figure S4: Ocean metagenome distribution of *Synechococcus* WH8102 PstS homologs.** Environmental abundance of (A) PstS1a, (B) PstS1b and (C) PstS2 homologs extracted from the Tara Oceans MetaG dataset (40,41). The blue-filled circle size denotes measured abundance at a particular sampling site. Abundance is plotted for surface water only. Sampling sites are represented by ‘X’. The corresponding bubble plot for identified sequences across sampling depths SRF and surface waters is shown as a function of phosphate concentration. The Krona plot depicts the taxonomic distribution of PstS homolog hits selected to analyse metagenome abundance.

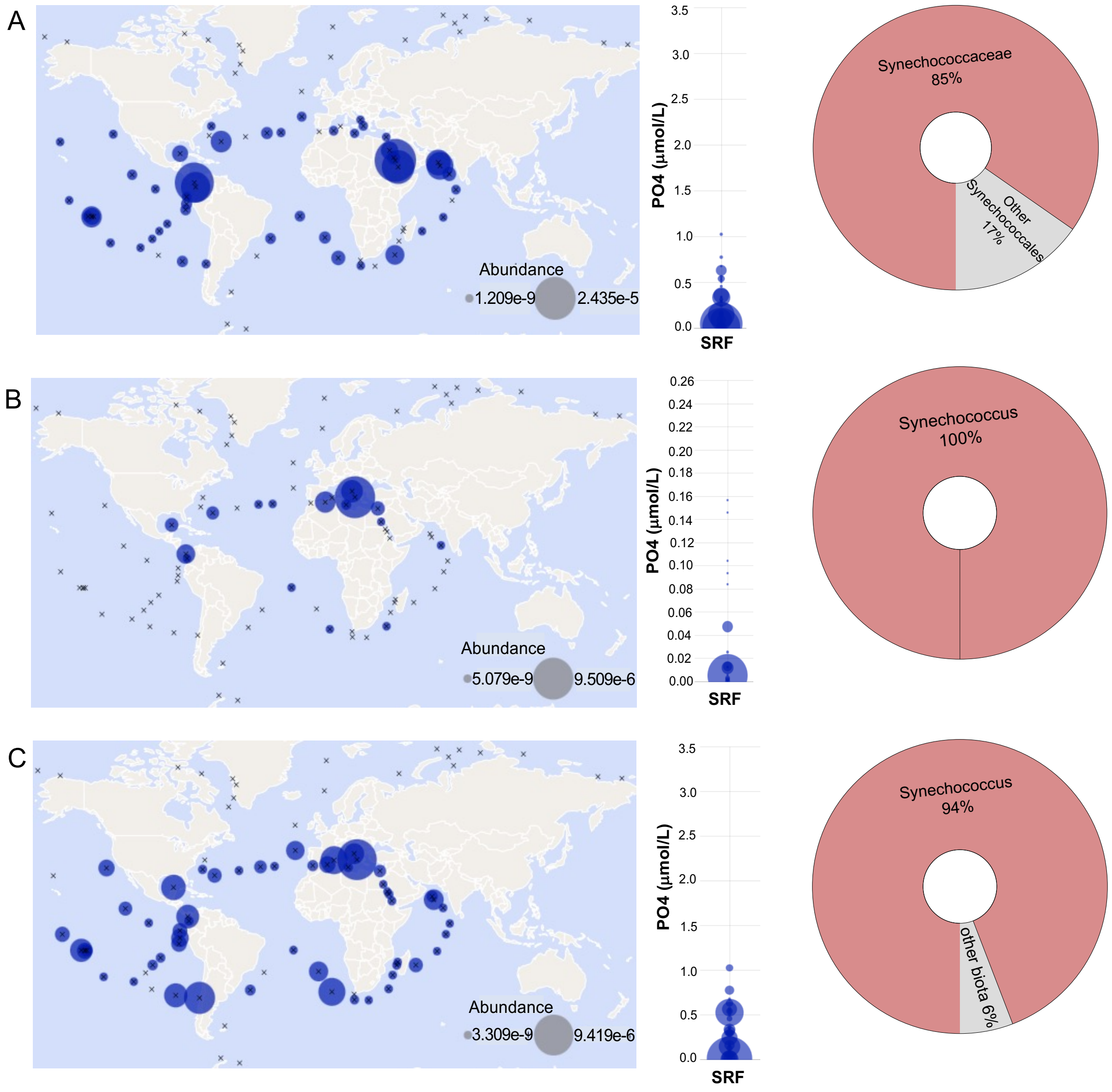

**Table S1: Amino acid sequence identity between PstS homologs from WH8102**

| | PstS1a | PstS1b |
|---|---|---|
| **PstS1a** | - | 73.33% |
| **PstS2** | 47.45% | 50.95% |

**Table S2: Signal P truncated mature PstS amino acid sequences**

| Target | Sequence |
|---|---|
| PstS1a | AEKLNGAGASFPAKIYQRWFADLAKSGGPQVNYQAVGSGSGRKAFI DQTVNFGASDDPMKKKDMAKVKRGVVQIPMVGGTIAFGYNKPGCD LKLTQEQAVRVAMGKIRNWQDLGCQPGTITWVHRSDGSGTTKAFT NSMQAFSSTWTLGTGKSVKWPAGVGAKGNSGVAGVIQNRMGAIGY VNQSYIKGKVVAAALQNKSGEFLKPSVAAGARALNGIQLDKDLAGK NPNPTAKGAYPIATLTWVLAYKTGNGKDAKVVQEAFNYMLSDAAQ DKAPSLGFVPLKGDILAKAKAAVNKIGE |
| PstS1b | SGRITGAGATFPAQIYQRWFGMLAGNDGPMVNYQAIGSGSGRKAYL DQTVNFGASDDPMISRDRRKVKRGVVQIPMIGGTIAFGYNKSGCELK LTQEQAVKVAMSAIKDWSELGCDPGPISWVHRSDGSGTTKAFTSSM AAFSSAWTLGTGKAVNWPSGVGAKGNAGVAAVIKKREGAIGYLNQ SYIRGSIKAAALQNLAGEFVKPSVEAGAIALNQITLDQNLAGENPNPS AAGAYPIATLTWVLAYERGNGPDAATIKDVFNFMLSDEAQNVAPRL GFVPLRGDILAKSKDAVNNIGE |
| PstS2 | SSGGSGGGDDKVTGKLNGAGASFPAAIYQRWFQELQPEGVTVNYQS VGSGAGVRQFMANTVDFGASDKPMKEAEIAKVERGVLQIPMTAGAI AVAYNLEGCDLKLTTEQLAGIFLGKIKNFSELGCADQKLTVVRRSDG SGTTYNFTKHLSAISEEWKNGPGAAKSIKWPTGVGSKGNEGVAAQL NQIPGGVGYVEAAYVKGKLQAAAVTNASGEQVKPTNETESTALDSI DIGPDLIGGNPNPPAGYPIVTFTWVLAYETGNGDKTAALKKTLEFML SEKAQSQAPELGYVSLPTGVVEKSLAAVEKISE |
